# Growth differentiation factor-15 (GDF-15) in localized pancreatic adenocarcinoma treated with multiagent chemotherapy: a biomarker analysis from the NEOLAP trial (AIO-PAK-0113)

**DOI:** 10.1016/j.esmogo.2025.100274

**Published:** 2025-12-15

**Authors:** B. Kimmel, S.T. Löhnert, F. Wedekink, M. Günther, S. Ormanns, I. Hartlapp, J. Siveke, G. Siegler, S. Boeck, H. Algül, U. Martens, F. Kullmann, T. Ettrich, S. Held, F. Anger, C.-T. Germer, V. Heinemann, J. Wischhusen, V. Kunzmann

**Affiliations:** 1 Department of Internal Medicine II, Medical Oncology, University Hospital Würzburg, Würzburg, Germany; 2 Comprehensive Cancer Center Mainfranken, University Hospital Würzburg, Würzburg, Germany; 3 Department of Gynecology, University Hospital Würzburg, Würzburg, Germany; 4 Innpath Institute of Pathology, Tirol Kliniken, Innsbruck, Austria; 5 Institut für Allgemeine Pathologie, Medizinische Universität Innsbruck, Innsbruck, Austria; 6 Department of Internal Medicine, St. Nikolaus-Stiftshospital GmbH, Akademisches Lehrkrankenhaus der Universität Bonn, Andernach, Germany; 7 Department of Medical Oncology, Bridge Institute of Experimental Tumor Therapy, West German Cancer Center, University Hospital Essen, Essen, Germany; 8 Division of Solid Tumor Translational Oncology, DKTK Partner Site Essen and German Cancer Research Center, DKFZ Heidelberg, Germany; 9 Department of Internal Medicine 5, Hematology and Medical Oncology, Paracelsus Medical University, Nürnberg, Germany; 10 Department of Medical Oncology and Comprehensive Cancer Center, University Hospital Grosshadern, LMU Munich, Munich, Germany; 11 Department of Hematology and Oncology, München Klinik Neuperlach, Munich, Germany; 12 Comprehensive Cancer Center München and Institute for Tumor Metabolism, TUM University Hospital, Klinikum rechts der Isar, Technical University of Munich, School of Medicine and Health, Munich, Bavaria, Germany; 13 Department of Internal Medicine III, SLK-Clinics Heilbronn GmbH, Heilbronn, Germany; 14 Department of Internal Medicine I, Kliniken Nordoberpfalz AG, Klinikum Weiden, Weiden, Germany; 15 Department of Internal Medicine I, Ulm University Hospital, Ulm, Germany; 16 Department of Biometrics, ClinAssess GmbH, Leverkusen, Germany; 17 Department of General, Visceral, Transplantation, Vascular and Pediatric Surgery, University Hospital Würzburg, Würzburg, Germany

**Keywords:** GDF-15, localized pancreatic adenocarcinoma, multiagent induction chemotherapy

## Abstract

**Background:**

The prognostic and predictive role of growth differentiation factor-15 (GDF-15) in localized, non-metastatic pancreatic ductal adenocarcinoma (PDAC) has not yet been explored.

**Patients and methods:**

During the prospective randomized phase II NEOLAP-1 (AIO-PAK-0113) trial for patients with therapy-naive locally advanced (borderline or unresectable) PDAC, blood (*n* = 131) and tumor tissue samples (*n* = 39) were collected. Using paired baseline and post-induction chemotherapy (ICT) samples, circulating GDF-15 (cGDF-15) levels were quantified by enzyme-linked immunosorbent assay, and local GDF-15 tumor expression (tGDF-15) was assessed by immunohistochemistry.

**Results:**

Lower baseline cGDF-15 levels (≤0.8 ng/ml) were significantly associated with increased median overall survival [21.92 (95% CI 19.73-24.16) versus 12.68 [95% confidence interval (CI) 10.56-14.81] months, *P* < 0.001, and significantly higher secondary R0 resection rates (36.5% versus 13.9%, *P* = 0.0051). In contrast to CA 19-9, cGDF-15 levels significantly increased after ICT [median 1.0 ng/ml [interquartile range (IQR) 0.62-1.5 ng/ml] at baseline versus median 2.37 ng/ml (IQR 1.32-4.43 ng/ml) at week 16], especially after treatment using platinum-based agents. In initial tumor specimens, GDF-15 expression was rare and predominantly confined to tumor cells. tGDF-15 correlated with high cGDF-15 levels at baseline [median 1.8 ng/ml (1.13-2.34 ng/ml) in positive tumor specimens, versus 0.76 ng/ml (0.55-1.17 ng/ml) in negative tumor specimens; *P* = 0.0087]. Similarly to cGDF-15, tGDF-15 expression increased after ICT (from 10% to 41% positive tumor specimens).

**Conclusions:**

High cGDF-15 levels at baseline are a negative prognostic and predictive biomarker in localized, non-metastatic PDAC. Considering that GDF-15 is further up-regulated by neoadjuvant multiagent chemotherapy, our data, together with recent findings on clinical effects of GDF-15, provide a strong rationale for upfront therapeutic GDF-15 blockade in localized PDAC.

## Highlights

• High baseline cGDF-15 is a negative prognostic and predictive biomarker in non-metastatic pancreatic ductal adenocarcinoma.
• GDF-15 is dynamically up-regulated during chemotherapy, especially platinum-based regimens.
• Our data provide a strong rationale for upfront therapeutic GDF-15 blockade in PDAC.

## Introduction

A major cause of cancer-related death worldwide is pancreatic ductal adenocarcinoma (PDAC), which is predicted to become the second leading cause of cancer-related death by the year 2030.^1^ At the time of diagnosis, ∼25%-30% of patients present with non-metastatic, but locally advanced PDAC, in which probability of margin negative tumor resection is low and depends on the degree of contact between tumor and adjacent blood vessels and organs. Complete surgical resection remains the only potentially curative option for patients with non-metastatic PDAC.^2^ Either induction chemotherapy (ICT) with or without chemoradiation, or multiagent regimens such as fluorouracil, leucovorin, irinotecan, and oxaliplatin (FOLFIRINOX), or nab-paclitaxel plus gemcitabine resulted in improved R0 resection rates and improved median overall survival (OS).^3^, ^4^, ^5^

Growth differentiation factor-15 (GDF-15), also designated as macrophage inhibitory cytokine 1, is a divergent member of the transforming growth factor-β superfamily.^6^ In healthy individuals, GDF-15 expression is low (0.2-1.15 ng/ml), and most prominent in the placenta,^7^^,^^8^ but its expression is induced under stress and pathological conditions, including inflammation, cardiovascular diseases, obesity, and cancer.^9^^,^^10^

Increased GDF-15 serum levels were correlated with a poor prognosis in many advanced cancers. In patients with pancreatic cancer, higher serum levels of GDF-15 were found in advanced stages compared with benign tumors, chronic pancreatitis, and healthy controls.^11^, ^12^, ^13^, ^14^, ^15^, ^16^ GDF-15 was recently shown to interfere with antitumor immune response. GDF-15 blockade synergistically enhanced the efficacy of immune checkpoint inhibition in tumors with high GDF-15 expression.^17^^,^^18^ An inverse relationship between GDF-15 mRNA expression and T-cell transcriptomic signature could also be demonstrated for PDAC.^18^ In addition, GDF-15 also plays an important role in cancer cachexia.^19^ Most studies investigating the prognostic and predictive value of GDF-15 involved metastatic or advanced stages of cancer. The prospective randomized phase II NEOLAP trial carried out a systematic surgical exploration after 4 months of multiagent ICT (nab-paclitaxel/gemcitabine alone versus nab-paclitaxel/gemcitabine, followed by FOLFIRINOX) in patients with localized, non-metastatic pancreatic cancer (borderline or unresectable PDAC). The aim of the present study was to analyze the correlation of GDF-15 expression with OS and R0 resection rate in order to clarify the prognostic and predictive value of GDF-15 in non-metastatic PDAC conducted on data of the NEOLAP trial.

## Patients and methods

### Study design and patient population

In this study, circulating GDF-15 (cGDF-15) levels were measured at baseline and after ICT at week 16. Their predictive and prognostic value was analyzed for OS and R0 resection rate, based on data from the prospective randomized phase II NEOLAP-AIO-PAK-0113 trial (NCT02125136). Patient population and study design of the multicenter NEOLAP study were described in detail previously.^3^ In brief, at 28 academic and non-academic hospitals in Germany, 165 patients with histologically or cytologically confirmed locally advanced PDAC were enrolled and treated over 4 months with two different multiagent ICT regimens, either nab-paclitaxel plus gemcitabine alone (treatment arm A) or nab-paclitaxel/gemcitabine, followed by FOLFIRINOX (treatment arm B). Eligible patients were aged 18-75 years and with an Eastern Cooperative Oncology Group performance status of 0-1. After completion of ICT, all patients without disease progression underwent explorative laparotomy and, if applicable, complete tumor resection. Serum used for cGDF-15 analysis was drawn at timepoint baseline and at week 16 (±1 week post-ICT) and the concentrations of cGDF-15 were detected by enzyme-linked immunosorbent assay (ELISA) as described below. Available tumor specimens were collected before and after ICT and expression of tumor GDF-15 (tGDF-15) was assessed by immunohistochemistry as mentioned later in the text. Patients with missing cGDF-15 baseline levels were excluded from the current analysis. The study was approved by the ethics committee at each participating center and was carried out in accordance with the International Conference on Harmonisation Guideline requirements for Good Clinical Practice and the Declaration of Helsinki. All patients provided written informed consent before participation.

### GDF-15 enzyme-linked immunosorbent assay

Circulating GDF-15 (cGDF-15) levels were determined using ELISA. To circumvent the confounding by the GDF-15 H202D variant,^20^ the commercial R&D DuoSet kit (R&D Systems, Minneapolis, USA) was modified using an in-house-developed anti-GDF-15 antibody (patent EP3122775A1) for capture.

Serum was diluted 1 : 20. The assay can reliably quantify GDF-15 levels ≥0.1 ng/ml. In brief, 96-well microplates were coated overnight with 100 μl diluted capture antibody (2 μg/ml) per well, and were washed and blocked before the diluted samples and standards were applied for 2 hours at room temperature. After incubation, bound GDF-15 was detected with biotinylated anti-GDF-15 detection antibody, followed by streptavidin-horseradish peroxidase and 3,3′5,5′-tetramethylbenzidin. The color reaction was stopped by addition of H_2_SO_4_ (1 mol/l) and absorbance at 450 nm was recorded in an ELISA reader (Sunrise, Tecan).

### Immunohistochemistry (IHC)

GDF-15 expression was detected by IHC on 4 μm thick sections of formalin-fixed paraffin-embedded tumor material using a mouse monoclonal antibody (CL0328, Invitrogen, Carlsbad, CA, USA) at a 1 : 50 dilution on a DAKO Omnis autostainer instrument (DAKO, Glostrup, Denmark). Appropriate positive controls were included in each staining run (placental tissue). The expression strength and localization were examined independently by two pathologists (MG, SO), who were blinded to patient outcome. Tumors with ≥10% GDF-15-positive tumor cells were considered positive. Discrepant cases were discussed and agreement was reached. Microphotographs were acquired as described previously.^21^

### Statistical analysis

Data were analyzed using IBM SPSS Statistics Version 29.0.0.0 or GraphPad Prism 10. Descriptive data are shown as median with interquartile range (IQR) for continuous or total with percent for categorical parameters. GDF-15 response was analyzed in all patients of the GDF-15 cohort with post-ICT (week 16) GDF-15 measurement. Data were compared using a two-sided Fisher’s exact test or Mann–Whitney *U* test. *P*-values < 0.05 were considered statistically significant.

OS [expressed as median with 95% confidence interval (CI)] was calculated from the start of ICT using the Kaplan–Meier method for two different established cGDF-15 cut-off levels (≤/>0.8 ng/ml and ≤/>1.5 ng/ml). The used cut-off points were determined in the course of a previous study in the non-metastatic setting of malignant melanoma,^22^ and compared for statistical significance by a two-sided log-rank test. Patients who were alive at the last follow-up were censored. Hazard ratio (HR) with 95% CI was calculated by Cox regression analysis.

## Results

### Characterization and outcome of GDF-15 study population

From 165 patients enrolled in the NEOLAP-PAK-0113 study, 131 (79%) were available for cGDF-15 baseline measurements. They formed the GDF-15 population for this study. Baseline characteristics and outcome of the GDF-15 study population compared with the total NEOLAP study population are shown in Table 1. The median age of this population was 62 years (IQR 55-68 years). This was evenly distributed between 69 (52.7%) male and 62 (47.3%) female participants. In total, 30 (22.9%) patients were not eligible for randomization. During treatment, 51 (38.9%) patients were randomly assigned to treatment arm A (nab-paclitaxel plus gemcitabine group), and 50 (38.2%) patients to treatment arm B (sequential FOLFIRINOX group). A total of 81 patients from the GDF-15 study population completed ICT (arm A, *n* = 42; arm B, *n* = 35; not randomized, *n* = 4), and therefore were available for week 16 (post-ICT) GDF-15 analysis. Baseline characteristics and outcome parameters, such as resection rates and OS status, were comparable between the GDF-15 and the NEOLAP population, and thus considered representative of the whole NEOLAP study population. R0 resections were achieved in 22.9% (30/131) of the GDF-15 population and 22.4% (37/165) of all treated patients (Table 1). As already shown for the total NEOLAP study population^3^ or the CA 19-9 study population,^23^ only R0 resection was associated with a pivotal survival benefit in the GDF-15 study population [R0 versus non-R0 resected (R1 and not resected) patients: 40.21 (95% CI 27.05-53.37) months versus 13.78 (95% CI 11.26-16.3) months] (Supplementary Figure S1, available at https://doi.org/10.1016/j.esmogo.2025.100274).

**Table 1:** Baseline characteristics and outcome

	GDF-15 population *n* = 131	Total NEOLAP population *n* = 165
Baseline characteristics		
Median age, years (IQR)	62 (55-68)	63 (56-69)
Sex, *n* (%)		
Male	69 (52.7)	85 (51.5)
Female	62 (47.3)	80 (48.5)
Treatment arm, *n* (%)		
A: nab-paclitaxel/gemcitabine	51 (38.9)	64 (38.8)
B: sequential FOLFIRINOX	50 (38.2)	66 (40)
Not randomized	30 (22.9)	35 (21.2)
ECOG performance status, *n* (%)		
0	93 (71.0)	115 (69.7)
1	34 (26.0)	43 (26.1)
Missing	4 (3.1)	7 (4.2)
Median cGDF-15, ng/ml (IQR)	1.0 (0.62-1.5)	—
Median CA 19-9, U/ml (IQR)	280 (61.2-1075)	276 (70-988)
Outcome at week 16		
Resection status		
R0 resected	30 (22.9)	37 (22.4)
Non-R0 resected	101 (77.1)	128 (77.6)
Survival, mOS, months (95 CI)		
R0 resected	40.21 (27.05-53.37)	40.2 (21.0-59.4)
Non-R0 resected	13.78 (11.26-16.3)	14.0 (11.76-16.32)

CA 19-9, carbohydrate antigen 19-9; cGDF-15, circulating growth differentiation factor-15; CI, confidence interval; ECOG, Eastern Cooperative Oncology Group; IQR, interquartile range; mOS, median overall survival; non-R0 resected, R1 resection and not resected.

### Prognostic and predictive value of baseline circulating GDF-15 levels

**Figure 1:**
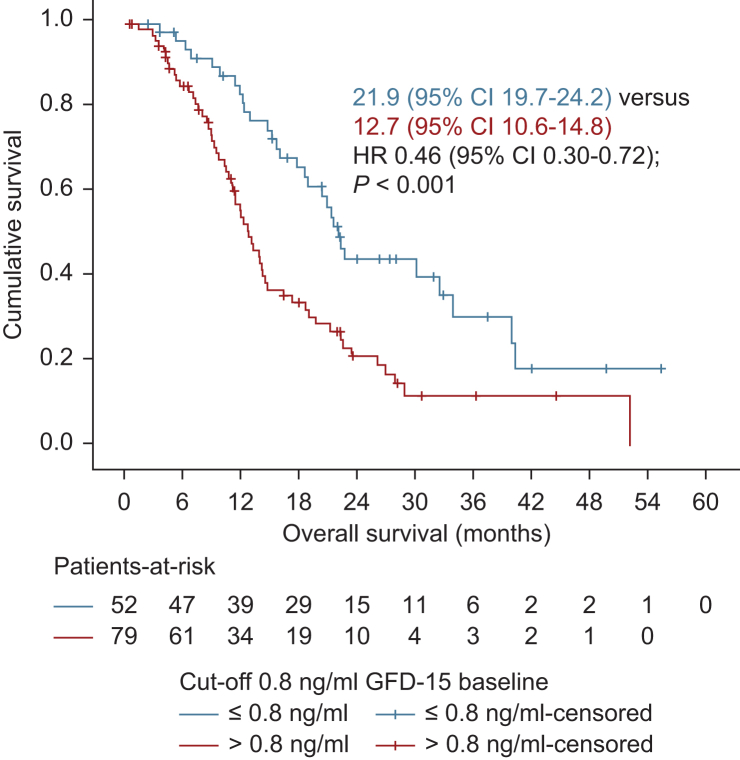
**Overall survival by circulating GDF-15 levels (cGDF-15).** Overall survival for patients with cGDF-15 levels ≤0.8 ng/ml (blue) versus >0.8 ng/ml (red) in the GDF-15 study population (*n* = 131). CI, confidence interval; HR, hazard ratio.

Baseline circulating GDF-15 (cGDF-15) levels in the study population (*n* = 131) ranged from 0.3 to 6.1 ng/ml [median 1.0 ng/ml (IQR 0.62-1.5 ng/ml)]. To evaluate the prognostic value of GDF-15, two different cut-off points of cGDF-15 at baseline were compared for OS in the entire GDF-15 population. As demonstrated in Figure 1, patients with baseline cGDF-15 ≤0.8 ng/ml showed a significant survival benefit compared with patients with cGDF-15 levels above 0.8 ng/ml. Median OS in the cGDF-15 group ≤0.8 ng/ml (*n* = 52) was 21.92 months (95% CI 19.73-24.16 months), versus 12.68 months (95% CI 10.56-14.81 months) in the >0.8 ng/ml cGDF-15 group (*n* = 79, *P* < 0.001). Baseline cGDF-15 levels below a higher cut-off (≤1.5 ng/ml, *n* = 99) were also correlated with a significant prolonged mOS [18.81 months (95% CI 13.98-23.64 months), as with 11.38 months (95% CI 8.95-13.8 months) in the cGDF-15 group >1.5 ng/ml (*n* = 32), *P* = 0.009, data not shown]. Furthermore, the prognostic impact of low cGDF-15 baseline levels was also present in the non-R0 cohort (*n* = 101), though to a lesser extent. Non resected patients or patients with R1 resection and baseline cGDF-15 levels ≤0.8 ng/ml (*n* = 33) had a significant survival benefit compared with patients with cGDF-15 levels above 0.8 ng/ml (*n* = 68). Median OS was 18.81 months (95% CI 14.76-22.85 months) versus 11.94 months (95% CI 9.99-13.88 months), respectively, HR 0.59 (95% CI 0.35-0.98), *P* = 0.039.

To evaluate the predictive value of baseline cGDF-15 levels for complete resection, the secondary R0 resection status was compared in patients with cGDF-15 levels >/<0.8 ng/ml (≤0.8 ng/ml, *n* = 52 or >0.8 ng/ml, *n* = 79) (Table 2). In the group with cGDF-15 baseline levels ≤0.8 ng/ml, 19 out of 52 patients received complete R0 resection (36.5%), whereas cGDF-15 baseline levels >0.8 ng/ml were accompanied with significantly lower R0 resection rates of 13.9% (11/79, *P* = 0.0051). The higher baseline cGDF-15 cut-off level (>/<1.5 ng/ml) also revealed a numerically higher secondary R0 resection rate in patients with lower cGDF-15 baseline levels [26.3% (26/99) versus 12.5% (4/32), respectively, *P* = 0.147].

**Table 2:** Correlation between circulating GDF-15 (cGDF-15) baseline levels and resection status

cGDF-15 at baseline	R0 resected	Non-R0 resected	*P* value∗
≤0.8 ng/ml	19/52 (36.5%)	33/52 (63.5%)	0.0051
>0.8 ng/ml	11/79 (13.9%)	68/79 (86.1%)	
≤1.5 ng/ml	26/99 (26.3%)	73/99 (73.7%)	0.147
>1.5 ng/ml	4/32 (12.5%)	28/32 (87.5%)	

cGDF-15, circulating growth differentiation factor-15; non-R0 resected, R1 resection and not resected.

∗ *P* value based on Fisher’s exact test.

### Dynamics of circulating GDF-15 levels during ICT

From the 81 patients completing ICT, 42 patients had been randomized to treatment arm A and 35 patients to treatment arm B (four patients were not randomized).

**Figure 2:**
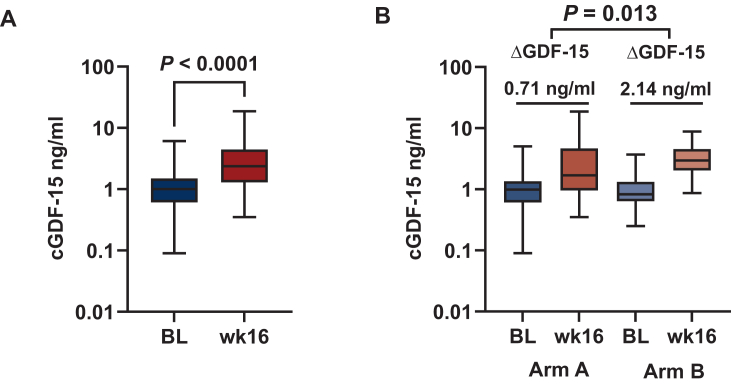
**Baseline (BL) and week 16 (wk 16) circulating GDF-15 (cGDF-15) levels**. (A) Increase of cGDF-15 levels in the total GDF-15 study population from median 1.0 ng/ml (IQR 0.62-1.5 ng/ml) at baseline (BL, *n* = 131) to median 2.37 ng/ml (IQR 1.32-4.43 ng/ml) after induction chemotherapy (ICT) at week 16 (wk 16, *n* = 81, *P* < 0.0001). (B) Analysis of cGDF-15 levels within the different treatment arms (group A: nab-paclitaxel/gemcitabine, *n* = 42; group B: nab-paclitaxel/gemcitabine followed by sequential FOLFIRINOX, *n* = 35) showed a significantly higher increase of cGDF-15 (ΔGDF-15) in arm B of 2.14 ng/ml versus 0.71 ng/ml in arm A, *P* = 0.013.

After ICT (week 16), cGDF-15 levels (*n* = 81) ranged from 0.35 to 18.25 ng/ml [median 2.37 ng/ml (1.32-4.43 ng/ml)], demonstrating a significant increase in cGDF-15 levels (*P* < 0.0001) from baseline [median 1.0 ng/ml (0.62-1.5 ng/ml)] to week 16 (Figure 2A).

As depicted in Figure 2B, in both treatment arms, either non-platinum-based ICT (nab-paclitaxel/gemcitabine alone, arm A) or platinum-based ICT (nab-paclitaxel/gemcitabine followed by FOLFIRINOX, arm B) exhibited a significant increase of cGDF-15 levels at week 16 compared with baseline levels [arm A: median baseline 0.985 ng/ml (0.61-1.35 ng/ml), versus week 16 1.695 ng/ml (0.96-4.66 ng/ml), *P* = 0.002; arm B: median baseline 0.83 ng/ml (0.64-1.32 ng/ml), versus week 16 2.97 ng/ml (2.04-4.56 ng/ml), *P* < 0.001]. The difference in cGDF-15 levels between baseline and week 16 (ΔGDF-15) within the treatment arms, however, showed a significantly higher increase of cGDF-15 levels in platinum-based arm B compared with non-platinum-based arm A (2.14 ng/ml versus 0.71 ng/ml, respectively, *P* = 0.013).

Patients with baseline cGDF-15 levels above 0.8 ng/ml and increasing cGDF-15 levels after ICT showed a trend of a worse OS compared with patients with stable or decreasing cGDF-15 levels [mOS 14.13 months (*n* = 29) versus 22.21 months (*n* = 13), respectively, *P* = 0.1798]. Dynamics of cGDF-15 during ICT were not correlated to R0 resection status, ORR (RECIST), or CA 19-9 response (data not shown).

### Correlation between circulating cGDF-15 and CA 19-9 levels before and after ICT

**Figure 3:**
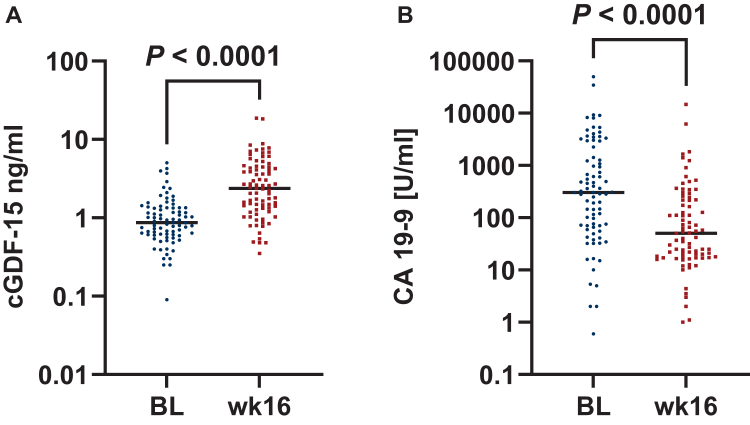
**Dynamics of cGDF-15 and CA 19-9 levels after induction chemotherapy (*n* = 81)**. Significant increase of cGDF-15 from baseline (BL) median 0.87 ng/ml (IQR 0.61-1.34 ng/ml) to median 2.37 ng/ml (IQR 1.32-4.43 ng/ml) at week 16 (wk 16) as compared with a significant decrease of CA 19-9 from median 303.5 U (IQR 68-1838 U) to median 50.4 U/ml (IQR 17.9-218.5 U/ml).

To correlate cGDF-15 with tumor burden, cGDF-15 was compared with CA 19-9, which is the only established prognostic and predictive circulating biomarker in PDAC. Both post-ICT cGDF-15 and CA 19-9 values were available for 81 patients. Baseline cGDF-15 levels were not correlated to baseline CA 19-9 levels, and therefore do not seem to be associated with baseline tumor burden (Supplementary Figure S2, available at https://doi.org/10.1016/j.esmogo.2025.100274). After ICT, a significant decrease in CA 19-9 levels from baseline to week 16 [303.5 U/ml (68-1838 U/ml) to 50.4 U/ml (17.9-218.5 U/ml); *P* < 0.0001] was demonstrated in the majority of patients indicating response to treatment. Conversely, as shown in Figure 3, cGDF-15 levels increased significantly [0.87 ng/ml (0.61-1.34 ng/ml), versus 2.37 ng/ml (1.32-4.43 ng/ml); *P* < 0.0001]. The difference in cGDF-15 levels between baseline and week 16 (ΔGDF-15) was not significant between patients with or without absolute decrease of CA 19-9 to values ≤50 U/ml (ΔGDF-15 1.76 ng/ml versus 1.26 ng/ml, *P* = 0.211), however. Similarly, patients with or without a relative CA 19-9 decrease of >50% also showed no significantly different ΔGDF-15 (1.54 ng/ml versus 1.45 ng/ml, *P* = 0.947), indicating no correlation between tumor response (reduction of tumor mass) and increase in cGDF-15 levels (Supplementary Figure S3, available at https://doi.org/10.1016/j.esmogo.2025.100274).

### GDF-15 expression in tumor specimens

**Figure 4:**
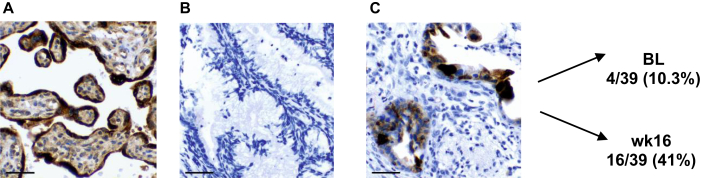
**GDF-15 expression in tumor specimens (tGDF-15).** (A-C) Exemplary immunohistochemical (IHC) staining for tGDF-15. (A) Placenta (= positive control), (B) negative (isotype control) and (C) representative positive GDF-15 staining in pancreatic tumor specimens. At baseline (BL), 4/39 (10.3%) specimens showed tGDF-15 expression compared with 16/39 (41%) after induction therapy (wk 16). Scale bar 200 μm.

To further determine the source and expression patterns of GDF-15, 39 available pairs of tumor specimens from two different time points (before and after ICT) were assessed by immunohistochemistry (IHC) (Figure 4). Out of these 39 patients, only 10.3% (4/39) showed a positive staining for GDF-15 (tGDF-15) before ICT, whereas in the majority (35/39) of patients, no relevant expression of tGDF-15 could be detected at baseline. As pictured exemplary in Figure 4C, tGDF-15 expression was confined to tumor cells and no relevant tGDF-15 expression was observed in the tumor microenvironment.

Median cGDF-15 levels in patients with positive IHC staining were 1.8 ng/ml (1.13-2.34 ng/ml) compared with 0.76 ng/ml (0.55-1.17 ng/ml) in patients with negative staining (*P* = 0.0087, Supplementary Figure S4, available at https://doi.org/10.1016/j.esmogo.2025.100274). Furthermore, all patients with a positive IHC result (tGDF-15-positive) before ICT had baseline cGDF-15 levels >0.8 ng/ml (4/4, 100%), whereas the proportion of patients with no detectable baseline tGDF-15 and higher cGDF-15 levels (>0.8 ng/ml) was only 42.9% (15/35).

After ICT, concordant to the observed increase in cGDF-15 levels, 16 out of 39 patients showed positive tGDF-15 expression, demonstrating an increase of tGDF-15-positive tumor specimens from 10% to 41%. Conversion of negative to positive detection of tGDF-15 correlated in 12 out of 13 (92.3%) patients with increased cGDF-15 levels, whereas only one patient (1/13) showed a decreased cGDF-15 level (Supplementary Figure S5A, available at https://doi.org/10.1016/j.esmogo.2025.100274). Interestingly, patients with conversion of negative to positive tGDF-15 expression after ICT in treatment arm B (platinum-based treatment) showed higher levels of cGDF-15 than patients in the non-platinum-based treatment arm A (Supplementary Figure S5B, available at https://doi.org/10.1016/j.esmogo.2025.100274). Dynamics of tGDF-15 expression during ICT were, similarly to cGDF-15 levels, not correlated to R0 resection status, ORR (RECIST), or CA 19-9 response (data not shown).

## Discussion

In this study, the comprehensive assessment of circulating and tumor-expressed GDF-15 (cGDF-15 and tGDF-15) in patients with localized, non-metastatic PDAC undergoing multiagent ICT has provided insights into its prognostic and predictive value. Our data demonstrate significantly better survival in patients with lower baseline cGDF-15 levels and, furthermore, a significantly higher secondary R0 resection rate compared with those with elevated cGDF-15 baseline levels. These findings suggest that baseline cGDF-15 is a valid prognostic and predictive biomarker in locally advanced PDAC before multiagent ICT.

Several studies reported that GDF-15 is overexpressed in cancer tissue as well as in serum samples in patients with PDAC compared with patients with benign pancreatic neoplasms, chronic pancreatitis, or healthy controls.^13^^,^^15^ O’Neill and coworkers examined the predictive capacity of circulating GDF-15 in asymptomatic individuals with a genetic and familial predisposition of pancreatic cancer and found elevated baseline levels in patients with neoplastic tumors compared with benign lesions.^24^ In our study, and to our knowledge for the first time, lower pretreatment cGDF-15 levels in PDAC patients were linked both to a survival benefit and the probability of R0 resection, underscoring the clinical utility of GDF-15 as a potent tool for treatment planning.

Notably, the value of baseline cGDF-15 as a prognostic biomarker appears, in this setting, stronger than that of baseline CA 19-9, as the latter failed to correlate with survival or resectability in this cohort, aligning with our previous correlative studies from the pivotal NEOLAP trial.^3^^,^^23^ Contrary to CA 19-9, cGDF-15 did not correlate with tumor burden or size, suggesting a function as a stress and immune-regulatory cytokine rather than a surrogate marker dependent on tumor mass.

The observed increase of cGDF-15 levels during chemotherapy, particularly in the platinum-based regimen, is a novel and clinically relevant finding in patients with localized, non-metastatic PDAC. While this up-regulation did not result in different resection rates or objective response, it may indicate a cellular stress response as a reaction to the cytotoxic effects of the chemotherapy or the selection of GDF-15-expressing tumor subclones. Also, induction of GDF-15 expression in stressed tumor-adjacent normal pancreatic tissue or extratumoral tissue cannot be ruled out.

Consistent with our data, Breen et al. reported higher cGDF-15 levels in patients with non-small-cell lung carcinoma, colorectal cancers, and ovarian cancers receiving platinum-based versus non platinum-based chemotherapy.^25^ Furthermore, in testicular cancer patients, GDF-15 levels increased during bleomycin, etoposide, cisplatin (BEP) chemotherapy, which correlated with endothelial damage markers and median baseline GDF-15 levels were not related to tumor stages.^26^ The simultaneous increase in tGDF-15, as shown by immunohistochemistry, further facilitates the idea that GDF-15 is dynamically regulated in response to cytotoxic therapy. Platinum-based therapy in particular causes extensive DNA damage, leading, among other things, to inhibition of DNA replication and activation of apoptosis.^27^ GDF-15 is substantially expressed in tumors and the tumor microenvironment and elevated levels are directly associated with the emergence and spread of malignancies.^10^^,^^28^ Tumor infiltrating fibroblasts can acquire permanent activated phenotype, leading to excessive production of ECM proteins, which accumulate and form fibrous connective tissues. This creates pressure within the tumor, known as solid stress. This stress induces fibroblasts to secrete GDF-15, which could facilitate migration of pancreatic cancer cells *in vitro*.^28^, ^29^, ^30^ In our study, baseline tGDF-15 expression was rare and seemed to be predominantly confined to tumor cells. However, Zabransky and coworkers also describe an age-dependent up-regulation of GDF-15 in cancer-associated fibroblasts in PDAC. Both tumor cells and stromal cells are suggested to be a source of GDF-15 after tumor formation.^31^ The observed GDF-15 induction by chemotherapy in this study warrants further investigation from additional preclinical and clinical studies, however.

Interestingly, while tGDF-15 was mostly absent in most tumors before treatment, its expression increased substantially (from 10% to 41%) post-ICT, similarly to circulating GDF-15 levels, suggesting that chemotherapy may reprogram tumor cells to up-regulate GDF-15 as a protective or adaptive mechanism. Recently, Haake and coworkers demonstrated that tumor-derived GDF-15 inhibits T-cell migration into tumor tissue and interferes with antitumor immune response. GDF-15 blockade with neutralizing antibodies enhanced both T-cell infiltration and the efficacy of anti-PD-1-mediated checkpoint inhibition in murine GDF-15-expressing tumor models.^17^

Additionally, an early clinical study further supported the immune suppressive role of GDF-15 in patients with non-squamous non-small-cell lung cancer and urothelial cancer. The combination of neutralizing anti-GDF-15 and anti-PD-1 antibodies markedly improved cancer treatment.^18^ Patients with elevated cGDF-15 baseline levels also gained weight during anti-GDF-15 treatment, which is in line with the anti-cachectic effect of GDF-15 blockade.^19^

Our findings strongly support the exploration of GDF-15 as a target in PDAC from a therapeutic perspective, particularly given the dismal efficacy of immune-checkpoint inhibitors to date. An upfront GDF-15 blockade, before or in combination with chemotherapy and immune-checkpoint inhibition, could potentially improve both resectability and survival by either lessening early protumoral effects of GDF-15 or enhancing anti-tumor immunity.

Although this study has several strengths, such as the homogeneous study cohort, the prospective collection of biomaterials, and the robust survival data within a randomized trial setting, there are some limitations. Due to the small sample size of the study population, our results need validation in a larger cohort. In particular, the relatively small number of tissue samples (*n* = 39) available for IHC and consequential limited conclusion on tGDF-15 needs further independent assessment. Furthermore, in our study, cGDF-15 levels were analyzed only at two timepoints; at baseline and after ICT. In addition, to confirm its diagnostic potential, cGDF-15 levels should be monitored more often during treatment to find the optimal cut-off value for cGDF-15 in order to further confirm the role and source of GDF-15 influencing tumor progression like tumor growth, metastasis, or response to therapy. Its expression levels in tumor tissue and/or the tumor microenvironment should be studied in more detail.

In summary, our data provide novel insights into the prognostic and predictive role of GDF-15, as a new clinically relevant and non-invasive biomarker in localized, non-metastatic PDAC, and underscores the potential of therapeutic GDF-15 inhibition in PDAC. Its dynamic up-regulation during chemotherapy and potential role in therapy resistance, immune evasion, and cancer cachexia provide a compelling rationale for GDF-15-targeted therapeutic strategies in future PDAC clinical trials.
