# Supplementary material for: Growth differentiation factor-15 (GDF-15) in localized pancreatic adenocarcinoma treated with multiagent chemotherapy: a biomarker analysis from the NEOLAP trial (AIO-PAK-0113)
Source: ESMO Gastrointest Oncol. 2025 Dec 15;11:100274. doi: 10.1016/j.esmogo.2025.100274 (PMC13080808; doi:10.1016/j.esmogo.2025.100274)

**Supplementary Figure S1:** Overall survival by resection status for R0-resected (blue) versus non-R0-resected, including R1 and not resected, (red) patients in the GDF-15 study population (n = 131). CI, confidence intervall; HR, hazard ratio


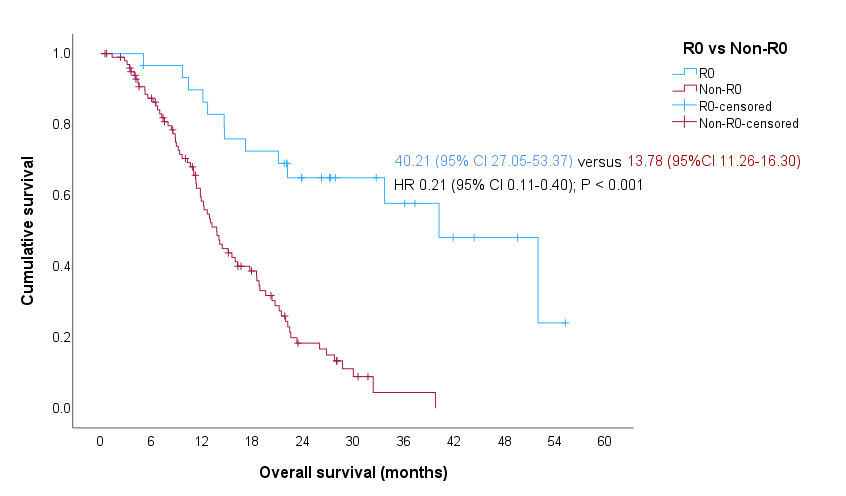


Patients-at-risk

| R0 | 30 | 28 | 26 | 21 | 14 | 10 | 8 | 4 | 3 | 1 | 0 |
| --- | --- | --- | --- | --- | --- | --- | --- | --- | --- | --- | --- |
| Non-R0 | 101 | 81 | 48 | 28 | 11 | 5 | 1 | 0 |  |  |  |

**Supplementary Figure S2:** Correlation between baseline cGDF-15 and baseline CA 19-9 levels.


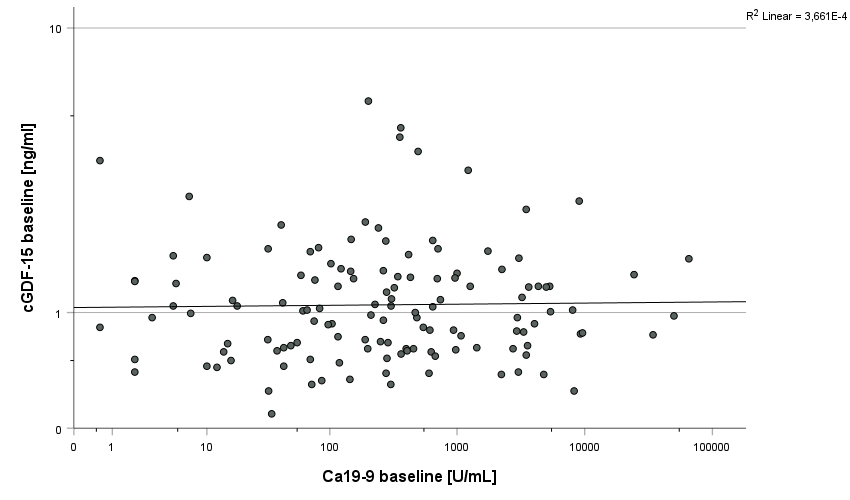


**Supplementary Figure S3:** Correlation between CA 19-9 reduction and ∆GDF-15 levels after induction chemotherapy. Neither absolute CA 19-9 reduction to values below 50 U/ml (A) nor relative CA 19-9 decrease > 50% (B) showed significant differences in cGDF-15 levels (∆GDF-15) between baseline (BL) and week 16 (wk16).

**B**

**A**

**P = 0.947**

**P = 0.211**

**∆GDF-15**

**1.26 ng/ml**

**∆GDF-15**

**1.54 ng/ml**

**∆GDF-15**

**1.45 ng/ml**

**∆GDF-15**

**1.76 ng/ml**

**CA 19-9**

**CA 19-9**

**> 50U/ml**

**≤ 50%**

**> 50%**

**≤ 50U/ml**

**Supplementary Figure S4**: Baseline cGDF-15 levels in patients either with negative (n = 35) or positive (n = 4) tGDF-15 expression in tumor specimens before therapy (P = 0.0087).


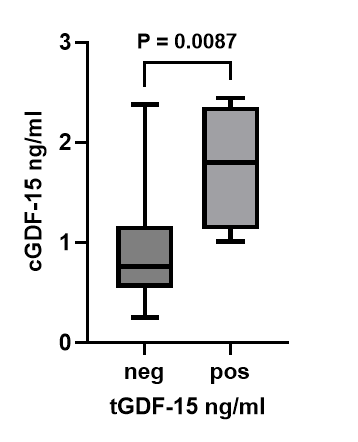


**Supplementary Figure S5**: Dynamics of circulating GDF-15 (cGDF-15) levels in patients with change of negative to positive GDF-15 expression in tumor tissue (tGDF-15) after induction chemotherapy (ICT). A: All evaluable patients (n = 13). After ICT (wk16), 12 of 13 patients (92.3%) with change of negative to positive tGDF-15 expression also showed increasing cGDF-15; B: Patients with non-platinum-based therapy (arm A) showed less increase of cGDF-15 compared to patients with platinum-based therapy (arm B).

**B**

**A**


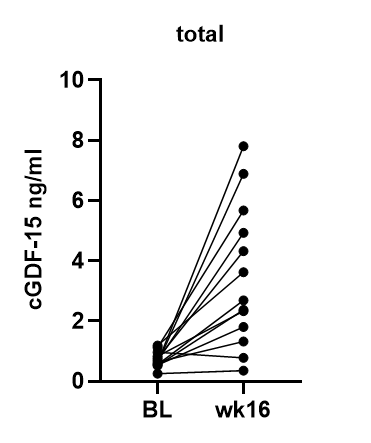

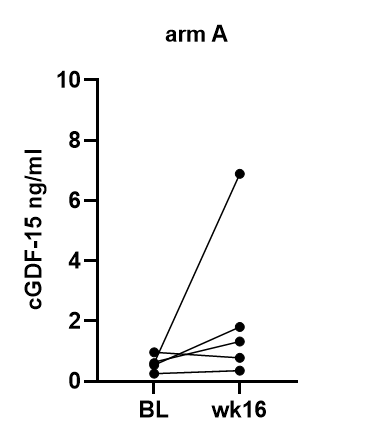

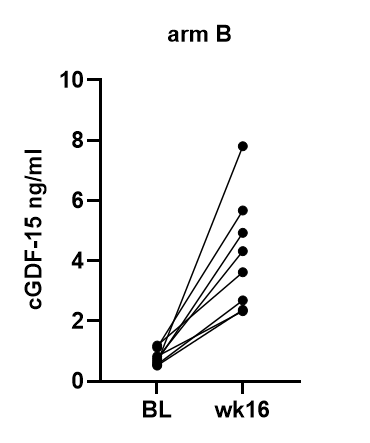

Supplement: Supplementary Figures [file mmc1.docx]
